# Down-regulation of ROCK2 alleviates ethanol-induced cerebral nerve injury partly by the suppression of the NF-κB signaling pathway

**DOI:** 10.1080/21655979.2020.1795404

**Published:** 2020-07-20

**Authors:** Xinguo Li, Jing Tong, Jihui Liu, Yibao Wang

**Affiliations:** a Department of Neurosurgery, The First Hospital of China Medical University, Shenyang, People’s Republic of China; b Department of Gastroenterology, The First Hospital of China Medical University, Shenyang, People’s Republic of China

**Keywords:** Ethanol, brain injury, apoptosis, oxidative stress, inflammatory response, NF-κB signaling pathway

## Abstract

Chronic alcohol consumption leads to hippocampal neuronal impairment, which related to neuronal death, oxidative stress, and inflammatory response. Rho-associated protein kinase 2 (ROCK2) is a major regulator in the central nervous system injury. However, the effects of ROCK2 in ethanol-induced brain injury have not been explored. In this work, we investigated the neuroprotective effects and the mechanism of ROCK2 inhibition *in vivo*. Wistar rats were exposed to 37% ethanol for 8 weeks to establish brain injury models. Morris water maze test was performed to evaluate cognitive function, and we found that the down-regulation of ROCK2 reduced the escape latency and increased the passing times and percentage of time spent in the target quadrant of rats. The results of H&E staining and Nissl staining showed that ROCK2 inhibition alleviated the pathological injury induced by ethanol. PI staining and Western blot confirmed that inhibiting ROCK2 attenuated the neuronal death and apoptosis as reflected by the reduced PI-positive neurons and the decreased expression of cleaved-caspase-3 and cleaved-caspase-9. Furthermore, the down-regulation of ROCK2 ameliorated the oxidative stress and inflammatory response induced by ethanol in rats as reflected by the up-regulation of IL-10, SOD, and GSH and reduction of TNF-α, IL-6, and MDA respectively. Additionally, Western blot and EMSA analysis revealed that the down-regulation of ROCK2 suppressed the nuclear transfer of NF-κB p65. In conclusion, our data suggested that ROCK2 inhibition ameliorated ethanol-mediated hippocampal neuronal impairment by anti-apoptotic, anti-inflammatory, anti-oxidative effects at least partially through the suppression of the NF-κB pathway.

## GraphicalAbstract

**Figure:**
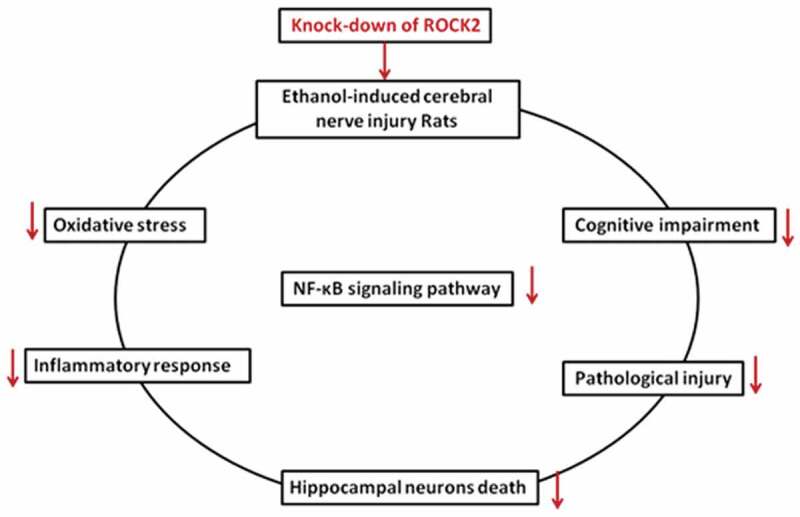

## 1. Introduction

In many societies, alcohol consumption is an integral part of daily life. The World Health Organization lists alcohol as one of the major causes of the global burden of disease in industrialized countries [1]. Chronic excessive drinking can cause a wide range of neurological disorders, including cognitive impairments such as learning and memory disorders [2]. Although the pathogenesis of alcohol has been studied extensively, little is known about the underlying protective mechanisms against its harmful effects [3].

Recent studies reported that alcohol consumption is related to neuroinflammation which may be a crucial cause of neuronal dysfunction [4]. Long-term exposure to ethanol leads to the activation of inflammasome in the brain [5]. Meisel et al. [6] found that ethanol triggers widespread apoptotic neurodegeneration in developing rat forebrain. Human monocytes isolated from brain tissues of healthy subjects and alcoholics after alcohol drinking also showed the evidence of apoptotic neuronal cell death [7]. Oxidative inflammation and neuronal apoptosis were shown to be involved in chronic alcohol-induced brain injury [8].

Recently, the role of gene therapy in neurodegenerative diseases has gradually attracted attention [9]. Rho-associated protein kinases (ROCK1 and ROCK2) are serine/threonine protein kinases [10]. Rho/ROCK is a major signaling pathway in the central nervous system [11]. Inhibition of ROCK protein in rodent models of spinal cord injury has been reported to promote axonal regeneration by reducing inflammation and neuronal apoptosis [12]. In addition, fasudil inhibitors improved spatial learning and memory impairment in Alzheimer’s rats via the deactivation of the NF-kB pathway [13,14], moreover, the inhibition of ROCK2 also restored neurological function in patients with ischemic stroke [15]. However, current research on ROCKs in alcoholic brain injury is not in-depth. ROCK2 has an enhanced expression in animal brains [16], but the role of ROCK2 in alcoholic brain injury has not been reported.

In the present work, we focused on the role of ROCK2 in ethanol-induced brain injury in rats. We found that inhibiting ROCK2 alleviated the cognitive dysfunction and pathological injury induced by ethanol in rats, and ROCK2 inhibition reduced the neuronal apoptosis, inflammatory response, and oxidative stress in rats partially through regulating NF-κB signaling pathway.

## 2. Materials and methods

### 2.1. Animals and treatments

Male Wistar rats (8 weeks, 280 ± 20 g, Liaoning Changsheng, Benxi, China) were allowed to acclimatize (12 h light/12 h dark cycles, 25 ± 1°C) for one week. The animal experiments were approved by the Ethics Committee of The First Hospital of China Medical University. A total of 96 rats were randomly divided into 4 groups, including normal control (Control) group, ethanol group, ethanol+ lentivirus-control (Lv-control) group, and ethanol + lentivirus-ROCK2 shRNA (Lv-ROCK2 shRNA) group, 24 rats in each group to ensure six biological repeats in each test. The ethanol-containing Lieber-DeCarli liquid diet was purchased from Dyets Inc. (Shanghai branch, Bethlehem, USA). As previously reported [17,18], rats in the ethanol treatment group received ethanol gradually increased from 0% to 11.8%, 23.6%, and finally to 37% in the first two weeks, so that the animals can gradually adapt to ethanol, then the ethanol proportion kept at 37% for 8 weeks. The pSico based control lentivirus or ROCK2 shRNA lentivirus was purchased from wanleibio (Shenyang, China). The rats were administrated with 1 × 10^8^ TU lentivirus at the beginning of the Lieber-DeCarli diet feeding via intravenous injection, then injected every four weeks at the same dose, three times in total. Rats in the NC group were given a control diet with maltose instead of alcohol.

### 2.2. Morris water maze

After 8 weeks of ethanol consumption, rats were used for the Morris water maze test. The experiment was performed in the water maze with a diameter of 180 cm circular pool filled with water (40 cm height). A platform (12 cm diameter) was located 2 cm below the surface of the water and was placed in the target quadrant. On the first day, rats were allowed to freely swim. On the second to the fourth day, rats were pre-trained to find the platform. Each rat was subjected to 4 trails per day in a maximum of 60 s on the fifth to the eighth day. In each trial, the time that rats climbed onto the hidden platform was recorded as escape latency. Next, on the ninth day, an probe trial was carried out to evaluate memory ability. For probe trial, the platform was removed, and the passing times of rats that crossed the previous location of the platform was recorded.

### 2.3. Nissl staining

The rats in each group were anesthetized, and the brain tissues were removed, fixed in 4% paraformaldehyde for 48 h. The tissues were cut into sections with 5 μm thickness. The sections were stained with 0.5% cresyl violet for 10 min and then were dehydrated in graded ethanol. The live neurons were evaluated by DP73 (OLYMPUS, Tokyo, Japan).

### 2.4. Hematoxylin and eosin (H&E) staining

The rat brains were isolated and then fixed by 4% paraformaldehyde. The tissues were frozen and cut into 5 μm-thick sections. Tissue processing was done as the standard procedure [19]. The sections were stained with H&E for histological evaluation.

### 2.5. Propidium iodide (PI) staining

Hippocampal neuronal apoptosis in rats was detected by the staining of PI. 4,6-diamino-2-phenyl indole (DAPI) was used to stain nuclei. Neurons were incubated with 15 μg/mL PI at room temperature for 1 h. The investigators were blinded for the treatments.

### 2.6. Inflammation and oxidative stress measurements

The levels of tumor necrosis factor-α (TNF-α) and Interleukin-6 (IL-6)in brain tissues of rats were detected by different ELISA kits (wanleibio, Shenyang, China), and the Interleukin-10 (IL-10) level in the brain was measured by ELISA kit (USCN KIT INC., Wuhan, China) following the manufacturer’s instructions.

The activities of superoxide dismutase (SOD) and the content of glutathione (GSH) and malondialdehyde (MDA) were evaluated by different detection kits (Nanjing Jian Cheng Bioengineering Institute, Nanjing, China) according to the manufacturer’s instructions.

### 2.7. Western blot

Proteins from brain tissues were extracted and quantified by bicinchoninic acid (BCA) protein assay kit (Solarbio, Beijing, China). Equal amounts of samples were separated by SDS-PAGE and transferred onto polyvinylidene fluoride (PVDF) membranes. The membranes were blocked with 5% skim milk for 1 h and incubated with the primary antibodies overnight at 4 ° C, including ROCK2 (1:1000, ABclonal, Wuhan, China), cleaved-caspase-3 (1:1000, Affinity, Changzhou, China), cleaved-caspase-9 (1:1000, Affinity, Changzhou, China), p-IκB (1:1000, Affinity, Changzhou, China), IκB (1:1000, Affinity, Changzhou, China), NF-κB (1:1000, ABclonal, Wuhan, China). β-actin (1:10,000, Santa Cruz, California, USA) and Histone H3 (1:5000, Gene Tex, Texas, USA) were used as internal controls. The membranes were incubated with goat anti-rabbit IgG-HRP or goat anti-mouse IgG-HRP secondary antibody (1:3000, Solarbio, Beijing, China) at 37 ° C for 1 h. The proteins were determined by an ECL western blot detection kit (Solarbio, Beijing, China).

### 2.8. Electrophoretic mobility shift assay (EMSA)

Nuclear protein was extracted from rat brain tissues and was analyzed using EMSA to determine NF-κB nuclear translocation. Three micrograms of nuclear protein were incubated with biotin-labeled double-stranded NF-κB binding consensus oligonucleotides (5'-AGTTGAGGGGACTTTCCCAGGC-3', Sangon, Shanghai, China) using Chemiluminescence EMSA kit (wanleibio, Shenyang, China). The binding reaction was performed at room temperature for 20 min. The DNA-protein complexes were electrophoresed on 6% non-denaturing polyacrylamide gels, transferred onto a nylon membrane (Millipore, Boston, Massachusetts, USA), and detected according to the manufacturer’s instructions.

### 2.9. Statistical analysis

Data were presented as mean ± SD. All statistical analyzes were carried out by GraphPad Prism 8.0. Statistical analysis was performed using Repeated Measures Two-Way ANOVA or One-Way ANOVA for individual comparisons between groups. p< 0.05 was considered statistically significant.

## 3. Results

### 3.1. Inhibition of ROCK2 ameliorated ethanol-induced cognitive impairment in rats

**Figure 1.**
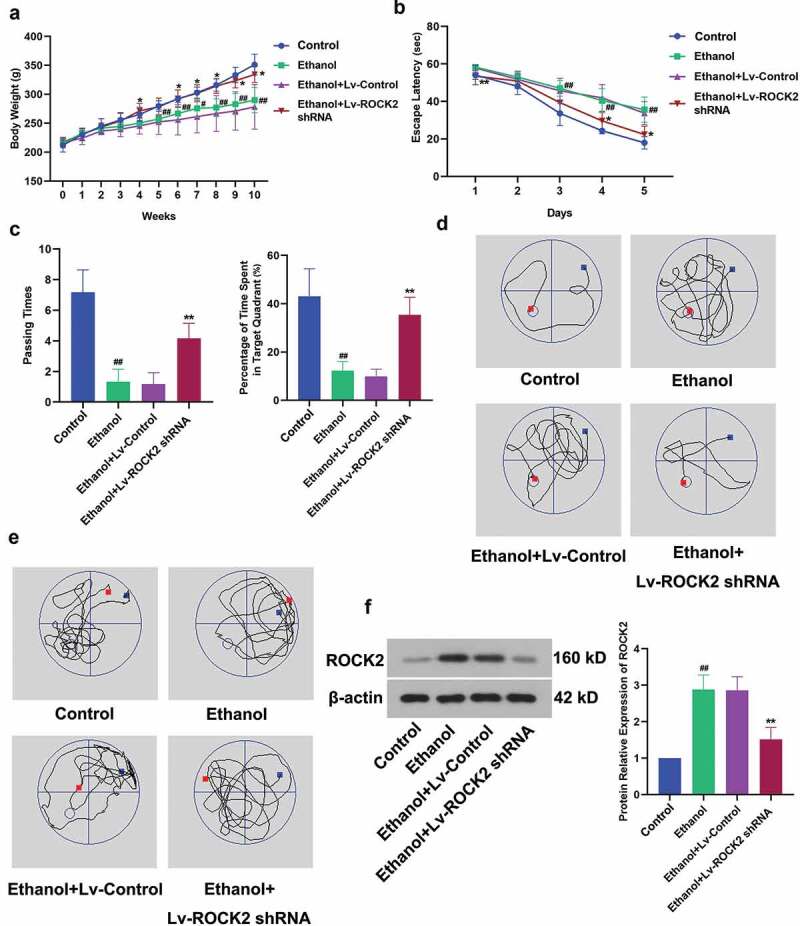
Effect of ROCK2 on body weight and ethanol-induced cognitive deficits in rats.

To explore the role of ROCK2 in ethanol-induced brain injury, rats exposed to ethanol were treated with lentivirus of ROCK2 shRNA. From the fifth week, rats in the ethanol group exhibited markedly lower body weight than that in the Control group. However, the down-regulation of ROCK2 significantly alleviated the bodyweight loss of rats (Figure 1a). The results of the Morris water maze test revealed that ethanol led to learning and spatial memory deficits in rats compared with the NC group. But ROCK2 inhibition dramatically restored these abilities, as indicated by the reduced escape latency, increased passing times and percentage of time spent in the target quadrant (Figure 1b–e). Figure 1f displayed the expression of ROCK2 in the brain tissues of rats from each group. These results suggested that ROCK2 was involved in ethanol-induced cognitive dysfunction of rats, and this impairment was alleviated when ROCK2 was inhibited.

a. Bodyweight changes of rats were recorded during 10 weeks.b. Escape latency of each rat was recorded from days 1 to 5.c. Passing times and the percentage of time spent in the target quadrant in spatial acquisition trailsd. The trajectory chart of navigation trial. The blue mark represented the place where the rat entered the water, and the red mark is where the rat exited the water.e. The trajectory chart of the probe trial. The blue mark represented the place where the rat entered the water, and the red mark is where the rat exited the water.f. The expression of ROCK2 in rats from each group were detected by Western blot. The data were normalized to the control β-actin.Data are expressed as mean± SD. ^#^p < 0.05 and ^##^p < 0.01 compared to Control group; *p < 0.05 and **p < 0.01 compared to Ethanol+Lv-control group.

### 3.2. Down-regulation of ROCK2 alleviates the pathological injury induced by ethanol

**Figure 2.**
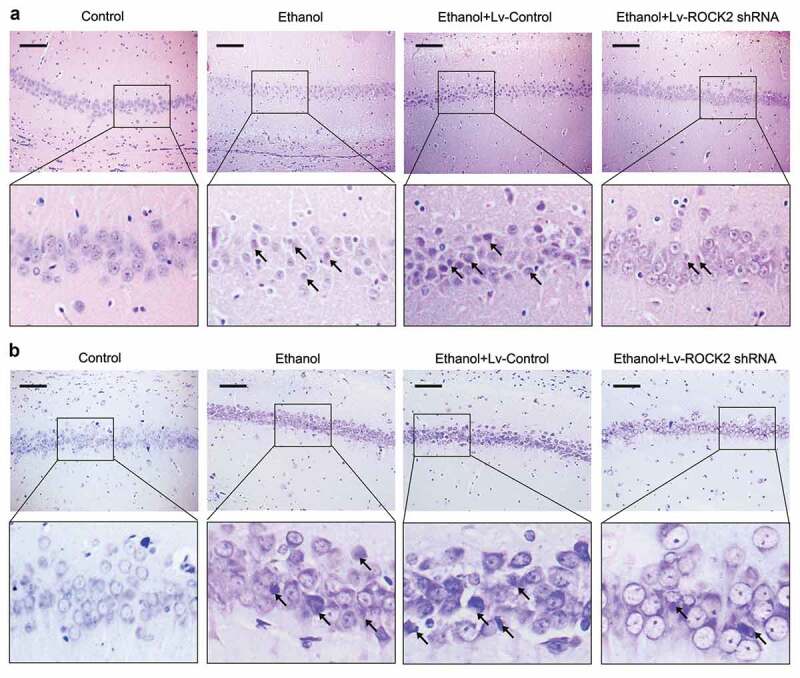
Down-regulation of ROCK2 alleviates the pathological injury of brain tissues in rats.

HE staining showed that control rats had clear arrangements of neurons in the hippocampus and complete cell structure, however, the neurons in ethanol-exposed rats showed an obviously shrinkage and deformation phenotype. These pathological changes were effectively alleviated by the inhibition of ROCK2 (Figure 2a). The results from Nissl staining further proved these pathological changes of the neurons in different groups (Figure 2b).

a. HE staining of brain tissues of rats from each group. Scale bar = 100 μm.b. Nissl staining for surviving neurons in the hippocampus of rats. Scale bar = 100 μm.The arrows indicates the typical damaged neurons.

### 3.3. Down-regulation of ROCK2 protected hippocampal neurons against ethanol-induced cell death in rats

**Figure 3.**
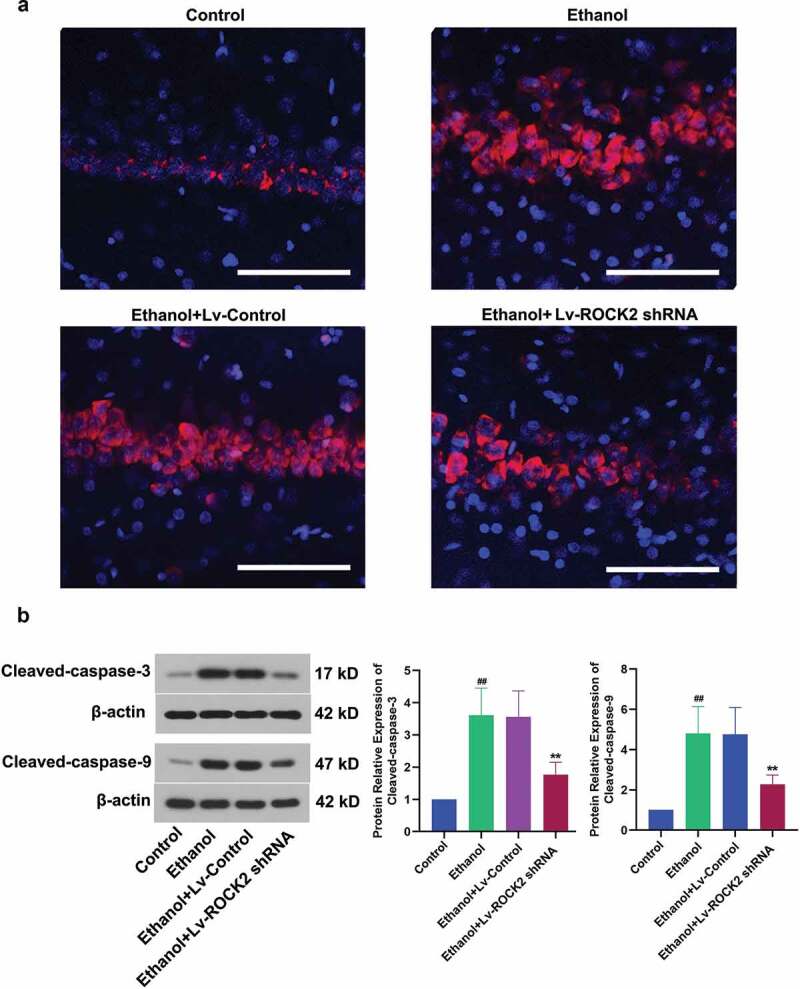
Down-regulation of ROCK2 protected hippocampal neurons against ethanol-induced cell death in rats.

To ascertain whether ROCK2 inhibition protected the rats from ethanol-induced neuron loss, the hippocampal tissues were further detected by PI staining. The results showed that PI was almost not incorporated into the neurons in the Control group, while ethanol stimulation caused increased PI-positive cells which indicted cell apoptosis. Furthermore, the PI-positive cells were markedly decreased by the down-regulation of ROCK2 (Figure 3a). Caspase-3 plays an important role in apoptotic cell death, and caspase-9 is a critical factor in the mitochondrial apoptosis pathway [20,21]. Our analysis indicated a significant increase in both cleaved-caspase-3 and cleaved-caspase-9 after ethanol treatment, while ROCK2 inhibition significantly reduced the cleaved-caspase-3 and cleaved-caspase-9 level (Figure 3b). Therefore, the down-regulation of ROCK2 could protect neurons from ethanol-induced apoptosis in rats.

a. PI-stained neurons were photographed. DAPI (blue) was used to stain the nuclei, PI (red) was used to stained dead neurons. Scale bar = 40 μm.b. The expression of cleaved-caspase-3 and cleaved-caspase-9 in brain tissues of rats from each group was measured by Western blot. β-actin was used as a loading control.Data are expressed as mean± SD. ^##^p < 0.01 compared to Control group; **p < 0.01 compared to Ethanol+Lv-control group.

### 3.4. Down-regulation of ROCK2 alleviates the inflammatory response and oxidative stress induced by ethanol in rats

**Figure 4.**
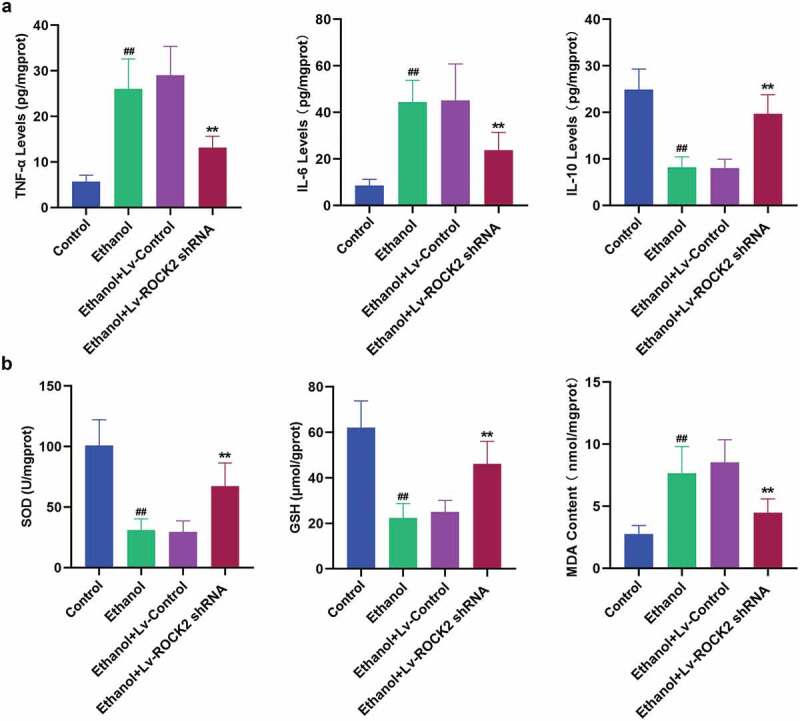
Down-regulation of ROCK2 alleviates the inflammatory response and oxidative stress induced by ethanol in rats.

To confirm whether ROCK2 inhibition reduced ethanol-induced inflammatory response, we examined the levels of inflammatory cytokines in the brain tissues of rats. Some cytokines, such as TNF-α and IL-6, have the effect of causing tissue cell damage and mediating inflammation. Our analysis revealed that the down-regulation of ROCK2 dramatically suppressed the ethanol-induced increase in the levels of TNF-α and IL-6 compared with the ethanol group (Figure 4a). The anti-inflammatory mediator, IL-10 was shown to antagonize inflammation-induced damage in the central nervous system [22]. ROCK2 shRNA treatment markedly enhanced the level of IL-10 in brain tissues of rats with comparison to the ethanol group (Figure 4a). To investigate whether ROCK2 inhibition protected the neuronal impairment through antioxidant mechanisms, the biomarkers of oxidative stress were evaluated. As indicated in Figure 4b, ethanol exposure led to reduced levels of SOD and GSH and an increased level of MDA, the index of lipid peroxidation, while treatment with ROCK2 shRNA remarkably reversed the above changes.

a. The levels of TNF-α, IL-6, and IL-10 in the brain tissues of rats from each group were measured by ELISAb. The effects of ROCK2 inhibition on the activities of SOD, GSH, and MDA from the different group was respectively detected.Data are expressed as mean± SD. ^##^p < 0.01 compared to Control group; **p < 0.01 compared to Ethanol+Lv-control group.

### 3.5. Down-regulation of ROCK2 suppresses the NF-κB signaling pathway induced by ethanol in rats

**Figure 5.**
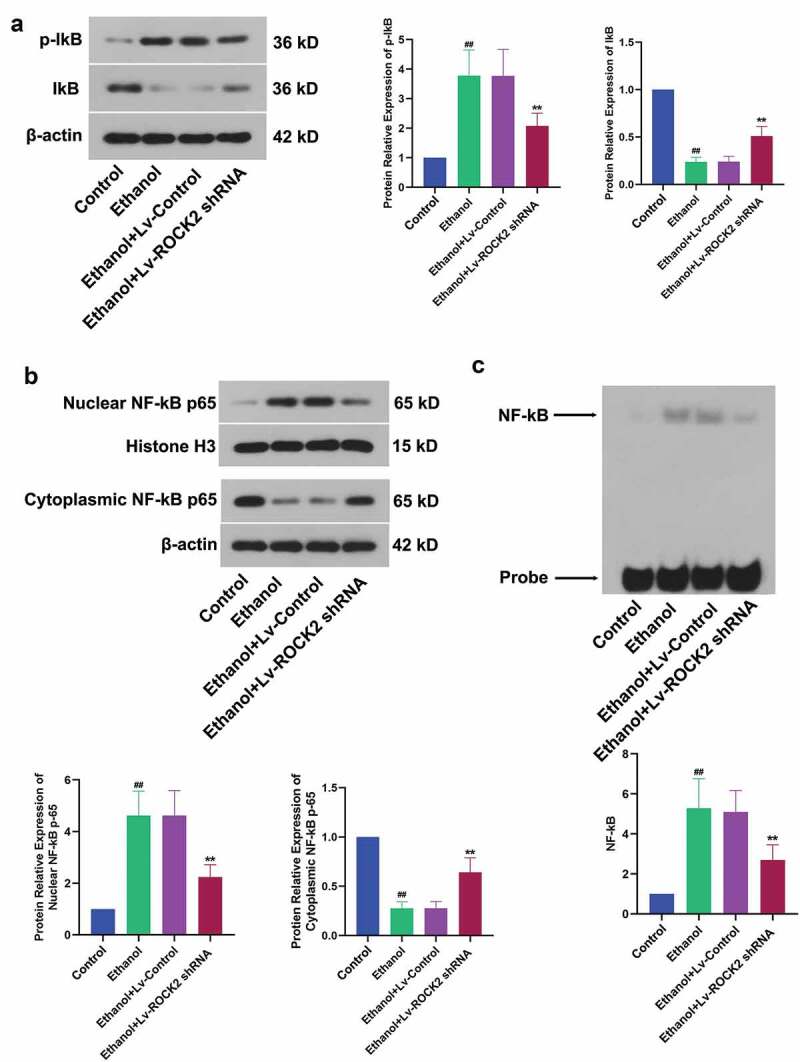
Down-regulation of ROCK2 suppresses the NF-κB signaling pathway induced by ethanol in rats.

NF-κB could be activated by inflammation, oxidative stress, and a variety of cytokines, and then participated in the regulation of gene expression in the process of inflammation. The ethanol-induced up-regulation of p-IkB and down-regulation of IkB, which indicated the inactivation of IκB kinase, was partially reversed by the treatment of ROCK2 shRNA (Figure 5a). At the same time, the activation of NF-kB induced by ethanol stimulation was also suppressed by ROCK2 inhibition (Figure 5b). In addition, we used EMSA to detect nuclear NF-κB DNA-binding activity. As expected, this binding was notably reduced after ROCK2 shRNA treatment in rats (Figure 5c). These results suggested that the down-regulation of ROCK2 suppressed the NF-κB signaling pathway.

a. The expression of p-IκB, IκB was determined by Western blot.b. The expression of nuclear NF-κB p65 and cytoplasmic NF-κB p65 was determined by Western blot.c. The activation of NF-κB was measured by EMSA.Data are expressed as mean± SD. ^##^p < 0.01 compared to Control group; **p < 0.01 compared to Ethanol+Lv-control group.

## 4. Discussion

Ethanol-induced brain injury is a chronic impairment of the frontal lobe and limbic system associated functions caused by long-term heavy drinking. It is characterized by a series of symptoms, such as slow response, memory loss, impaired mobility, personality change, irascibility, and dyspraxia. In this study, we demonstrated that down-regulation of ROCK2 improved ethanol-mediated cognitive dysfunction of rats, ameliorated pathological damage in the hippocampus, alleviated neuronal death, inflammation, and oxidative stress, which were likely related to the NF-κB signaling pathway.

ROCK2 is a major regulator of axonal degeneration, neuronal death, and axonal regeneration in the central nervous system [23]. The anti-apoptotic effects of ROCK2 inhibition on cells have been widely reported. Silencing ROCK2 significantly decreased the apoptosis of PC12 cells [24]. miR-335 inhibits apoptosis through targeting ROCK2 in acute ischemic stroke [25]. miR-582-5p inhibits the apoptosis of neuronal cells after cerebral ischemic stroke through regulating Rho/Rho-kinase signaling pathway [26]. Our data indicated that the down-regulation of ROCK2 dramatically attenuated the neuronal apoptosis induced by ethanol in rats, which was in line with the previous results.

The central nervous system is sensitive to oxidative damage due to its high oxygen demand and energy expenditure [27]. It has been reported that ethanol may cause tissue injury through lipid peroxidation [28], and exposure to ethanol for 4 weeks could induce oxidative stress [29]. Oxidative stress also caused hippocampal neuronal apoptosis [30], which led to cognitive dysfunction [31]. Therefore, it is critical to support the endogenous defense mechanism by the regulation of target genes. SOD is a natural superoxide free radical scavenging factor and is an important antioxidant enzyme, it can convert harmful superoxide radicals into hydrogen peroxide. GSH is an essential endogenous anti-oxidant in all animal cells, it protects from singlet oxygen and superoxide radicals and hydroxide radical damage through reacting with free radicals. MDA is a secondary product of lipid peroxidation and serves as an important index for measuring the degree of lipid peroxidation. In the present study, we found that ROCK2 inhibition reduced the oxidative stress induced by ethanol, as indicated by the increased levels of SOD and GSH and the reduced level of MDA. These findings suggested that ROCK2 inhibition alleviated ethanol-induced brain injury at least in part by inhibiting neuronal death and apoptosis and reduced oxidative stress.

Excessive oxidative stress can damage the antioxidant system in brain tissue and cause inflammation response, which can eventually lead to nerve cell death. Ethanol induction can activate microglia, which triggers inflammatory mediators in the brain and causes nerve damage [32]. The level of TNF-α was increased after brain injury, and its level was correlated to the degree of the injury [33], and IL-10 can inhibit TNF-α to reduce the inflammatory response after brain injury [34]. IL-6 is a pro-inflammatory cytokine that plays an important role in the central nervous system [35]. Previous reports showed that ROCK2 was activated in lung endothelial cells during inflammation [36], and the level of ROCK2 was significantly up-regulated in the inflamed mucosa from the patients with inflammatory bowel disease [37]. In this study, chronic exposure of ethanol increased the pro-inflammatory cytokines, TNF-α and IL-6, and reduced the anti-inflammatory cytokine, IL-10. However, the down-regulation of ROCK2 dramatically inhibited the neuroinflammation in ethanol-induced models.

NF-κB is a major signaling involved in regulating the inflammation-associated pathogenesis. Activation of NF-κB in the nervous system indicates inflammation, ischemia, injury, and other diseases in the brain [38]. NF-κB activation can enhance the pro-inflammatory cytokines transcription, meanwhile, the cytokines activate NF-κB in turn, which is known to amplify inflammatory signals [39]. NF-κB p65 subunit is usually sequestered by its inhibitor IκB in the cytoplasm. When activated by stimulation, it is rapidly translocated to the nucleus to induce a cascade of inflammatory response. It has been reported that the activity of NF-κB was increased in the rat brain cortex after brain injury [40]. NF-κB is closely associated with the pathological process of neuronal cell death [41] and related to neuroinflammation [42]. Some researchers found that after brain injury, rats lacking TNF-α receptors had fewer neurons damage, and the activity of NF-κB was also decreased [43]. To elucidate the potential mechanisms of the protective effects of ROCK2 inhibition, we explored the changes of IκB, p-IκB, and NF-κB p65 by Western blot. We found that the down-regulation of ROCK2 suppressed the translocation of NF-κB p65 from the cytoplasm to the nucleus in model rats. The results of EMSA further confirmed this conclusion. A prior study showed that inhibiting Rho-kinase suppressed apoptosis and inflammatory injury by regulating NF-κB activation in cisplatin-induced acute kidney injury [44], and our results are consistent with the previous study. Thus, down-regulation of ROCK2 could attenuate ethanol-induced brain injury at least partially through inhibiting NF-κB activation.

In addition to the NF-kB signaling pathway, previous study also suggested that ROCK2 is involved in LIMK2 mediated Aβ_42_-induced spine degeneration and neuronal hyperexcitability [45]. Specific knockdown of ROCK2 increased survival of retinal ganglion cells after optic nerve axotomy by the activation of AKT pathway [46]. Down-regulation of ROCK2 increased the expression of phospho-ERK1 and protected dopaminergic neurons in substantia nigra from 6-hydroxydopamine(6-OHDA)-induced degeneration [47]. Therefore, we speculate that down-regulation of ROCK2 may protect the nervous system form ethanol-induced injury by the regulation of LIMK2, AKT, ERK or other signaling pathway, we will investigate these in our further studies.

## 5. Conclusion

In a word, our data suggested the neuroprotective effects of ROCK2 inhibition on cognitive dysfunction and hippocampal neuronal impairment induced by ethanol in rats. This effect is associated with anti-apoptosis, anti-inflammation, anti-oxidation, as well as the suppression of the NF-κB signaling pathway. Our findings suggested that ROCK2 may be considered as a promising target in the future clinical therapy of alcohol-related brain injury.

## Abbreviations

H&E: hematoxylin and eosin
PI: propidium iodide
TNF-α: tumor necrosis factor-α
IL-6: interleukin-6
IL-10: interleukin-10
MDA: malondialdehyde
SOD: superoxide dismutase
GSH: glutathione
EMSA: electrophoretic mobility shift assay
NF-kB: nuclear factor kappa B
IkB: inhibitor kB
p-IkB: phosphorylated-IkB.

## Research highlights

- Inhibition of ROCK2 blocked the NF-kB pathway.
- Inhibition of ROCK2 alleviates ethanol-induced inflammation and oxidative stress.
- Inhibition of ROCK2 improves cognitive dysfunction.
